# Milled cereal straw accelerates earthworm (*Lumbricus terrestris*) growth more than selected organic amendments

**DOI:** 10.1016/j.apsoil.2016.12.006

**Published:** 2017-05

**Authors:** Tom Sizmur, Elodie Martin, Kevin Wagner, Emilie Parmentier, Chris Watts, Andrew P Whitmore

**Affiliations:** a Department of Sustainable Soils and Grassland Systems, Rothamsted Research, Harpenden, UK; b Department of Geography and Environmental Science, University of Reading, Reading, UK; c Ecole Supérieure d'Ingénieurs et de Techniciens pour l'Agriculture, Rouen, France; d Université de Poitiers, Poitiers, France

**Keywords:** Earthworm, Straw, Manure, Food, Energy, Ecosystem service

## Abstract

Earthworms benefit agriculture by providing several ecosystem services. Therefore, strategies to increase earthworm abundance and activity in agricultural soils should be identified, and encouraged. *Lumbricus terrestris* earthworms primarily feed on organic inputs to soils but it is not known which organic amendments are the most effective for increasing earthworm populations. We conducted earthworm surveys in the field and carried out experiments in single-earthworm microcosms to determine the optimum food source for increasing earthworm biomass using a selection of crop residues and organic wastes available to agriculture. We found that although farmyard manure increased earthworm populations more than cereal straw in the field, straw increased earthworm biomass more than manures when milled and applied to microcosms. Earthworm growth rates were positively correlated with the calorific value of the amendment and straw had a much higher calorific value than farmyard manure, greenwaste compost, or anaerobic digestate. Reducing the particle size of straw by milling to <3 mm made the energy in the straw more accessible to earthworms. The benefits and barriers to applying milled straw to arable soils in the field are discussed.

## Highlights

• Farmyard manure increased earthworm populations more than straw in the field.
• Milled straw increased earthworm growth more than milled manures in microcosms.
• Earthworm growth rates are positively correlated with amendment calorific value.
• Straw only benefits earthworm growth when milled.
• Incorporating milled straw in arable soils may increase earthworm populations.

## 1 Introduction

Earthworms are the most abundant animal, by biomass, in most soils (Lavelle and Spain, 2001) and are responsible for providing numerous ecosystem services and functions (Blouin et al., 2013) that benefit crop growth (Bertrand et al., 2015). Earthworms increase the rate of water infiltration (Bouché and Al-Addan, 1997), the availability of nutrients (Devliegher and Verstraete, 1996), and can increase crop yield by 25% (van Groenigen et al., 2014). Many agricultural practices such as tillage (Chan, 2001), pesticide application (Pelosi et al., 2014), and the removal of crop residues (Karlen et al., 1994) decrease the biomass and abundance of earthworm populations. Conversely, the addition of organic amendments to soils increases earthworm populations in arable soils (Edwards and Lofty, 1982), even when tillage operations and pesticide applications are maintained (Blanchet et al., 2016, Whalen et al., 1998).

Earthworm population dynamics can be explained by modelling the energy budgets of individuals within a population, and the interactions between the individuals (Jager et al., 2006, Johnston et al., 2014a, Johnston et al., 2014b). The models describe how individuals acquire and utilize energy, based on a set of simple rules for metabolic organisation, treating individual earthworms as a system with a closed mass and energy balance. Earthworms must reach a minimum mass to mature sexually and be able to reproduce (Lofs-Holmin, 1983). The quantity of food supplied (assuming all else is equal) also influences its reproduction rate because it converts food into offspring (Johnston et al., 2014b). It is possible to reduce the time taken for earthworms to reach maturity and intensively rear earthworm communities in laboratory cultures by optimising population density, temperature and moisture (Butt et al., 1992, Lowe and Butt, 2007, Lowe and Butt, 2005). However, these parameters cannot be easily manipulated in field populations.

The quality of food fed to laboratory reared earthworms affects earthworm biomass, time taken to reach sexual maturity and cocoon production (Butt, 2011). There is also considerable evidence that the abundance and biomass of earthworms in arable fields can be increased by the application of organic amendments such as straw (Kennedy et al., 2013), poplar bark (Pérès et al., 1998) and cattle slurry (Pommeresche and Løes, 2009). Reducing the particle size of organic amendments to < 2 mm increases the growth rate of laboratory-reared earthworms (Boström and Lofs-Holmin, 1986, Lowe and Butt, 2003). However, growth rate can differ to a large extent depending on the type of organic amendment applied. For example, livestock manures increase earthworm populations more than composts, reportedly because the organic carbon in the composts is more humified and stable due to microbial degradation (Leroy et al., 2008). However, despite crop residues (e.g. cereal straw) being less humified and less degraded by microorganisms at the time they are incorporated into the soil, they do not seem to increase earthworm biomass to the same extent as livestock manures (Blanchet et al., 2016).

In the UK, and many other nations, the availability of animal manures to cereal growers for land application is limited because of the geographical distance between livestock and arable farms, as evidenced by lower use of farmyard manure in the Eastern region (13% of crop and grass area), compared to the South West region (41% of crop and grass area) (DEFRA, 2016). Therefore, we investigated ways of increasing earthworm populations using cereal straw produced on most arable farms and contemporary soil amendments that are becoming increasingly available in arable regions (compost and anaerobic digestate). We hypothesised that earthworm biomass could be increased in soils by manipulating the type(s) of organic amendment(s) applied and their particle size.

## 2 Materials and methods

### 2.1 Field surveys

Earthworm surveys were carried out on two long term field experiments at Rothamsted Experimental Farm near Harpenden, UK (51.813N, 0.381 E) during spring 2014. All 16 plots of the Long Term Straw Incorporation Experiment, described by Powlson et al. (2011) were surveyed. The experiment has grown winter wheat continuously and had wheat straw incorporated annually for 28 years at a rate of none, once, twice, and four times the yield of straw the previous year (approximately 0, 5, 10 and 20 t ha^−1^) in a complete randomised block design (Table 1). A 2 m × 3 m area was designated specifically for sampling on the southern end of each plot. Two earthworm surveys were conducted in each plot (as described below), resulting in 32 surveys in total.

Selected plots on the Broadbalk experiment, described by Blair et al. (2006), that have grown winter wheat continuously for 171 years (apart from occasional fallow years) were also surveyed but, due to the age of the experiment, treatments are not replicated. Surveys were conducted on four plots that have either (i) received 35 t ha^−1^ of farmyard manure annually for 171 years, (ii) received wheat straw for the last 28 years by incorporating the straw of the previous crop harvested from the same plot (approximately 5 t ha^−1^), (iii) received both farmyard manure and wheat straw annually, as described above, or (iv) received no manure or straw applications for at least 171 years. All plots received 144 kg N ha^−1^ since 1852. A 1 m × 14 m area was designated specifically for sampling along the northern edge of each plot and this area was divided into four equal sub-plots that are considered here statistically as true replicates (Table 1). In each sub-plot two earthworm surveys were conducted, resulting in 32 surveys in total.

Earthworm surveys were conducted by excavating a 20 × 20 × 20 cm cube of soil, bringing it back to the on-site laboratory and sorting it to find all the earthworms and identify them following (Sherlock, 2012). Deep burrowing (anecic) earthworms were extracted by pouring a 5 L aqueous solution containing 6 g l^−1^ of Colman’s mustard flour, following (Bartlett et al., 2008, Murchie and Gordon, 2013) into the excavated hole and waiting up to 1 h to collect any emerging earthworms. All earthworms were washed by submerging them in water, blotted dry, identified to the species level and then its mass determined. All adults and some juveniles were identified but if the species of a juvenile earthworm was unclear then it was classified as ‘unidentified’.

### 2.2 Microcosm experiments

#### 2.2.1 Materials

A silty clay loam soil of the Batcombe Series (Avery and Catt, 1995), a Chromic Luvisol according to FAO classification, was collected from Fosters field of Rothamsted Experimental Farm. Fosters field has been in continuous arable production for more than 200 years. and has a soil organic carbon content of 14.3 g kg^−1^ (Johnston et al., 2009). The soil was air dried and sieved to <2 mm.

**Fig. 1:**
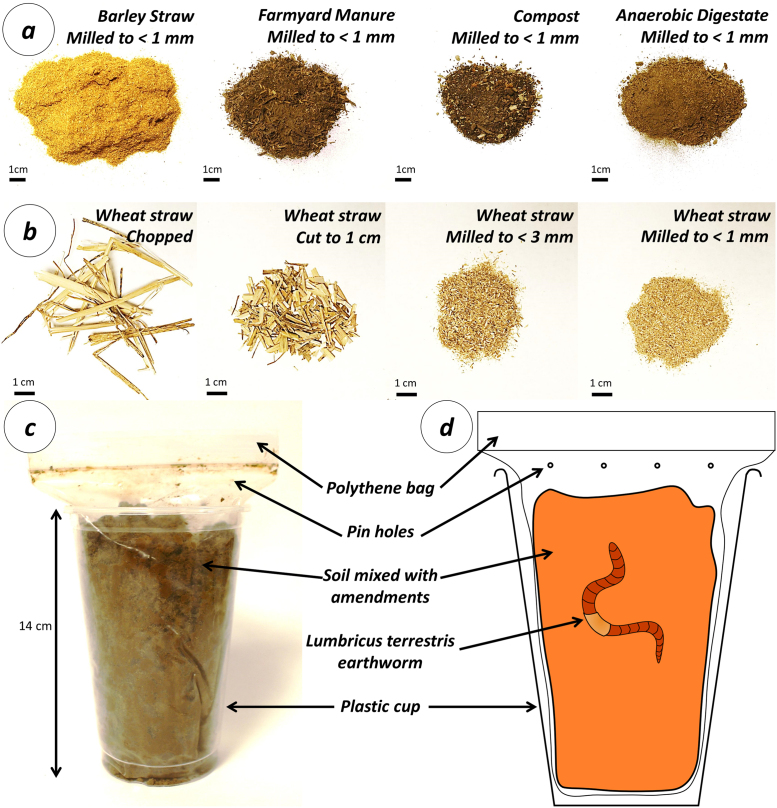
Amendments and experimental microcosms. Photographs (a) of barley straw, farmyard manure, compost and anaerobic digestate after milling to <1 mm and (b) wheat straw after chopping, cutting to 1 cm, milling to <3 mm and milling to <1 mm. Scale bars indicate 1 cm. Photograph (c) and schematic (d) of the experimental setup of microcosoms for determining the effect of amendments on changes in earthworm biomass.

Barley and wheat straw was also sourced from Rothamsted Experimental Farm. Farmyard manure was obtained from a farm with a mixed single suckling beef herd that is housed inside during the winter in bullock yards. Greenwaste compost was obtained from Organic Recycling Ltd. Anaerobic digestate was obtained from Staples Vegetables Ltd. and comprises the fibre portion of a brassica waste and maize-fed digester. All organic amendments were sampled shortly after delivery and air dried prior to being milled, to the sizes described below, using a Christy Turner Lab Mill and a <1 mm sample analysed for N and C concentration using a LECO TruMac Combustion Analyser, and for gross energy by Sciantec Analytical Services Ltd. using a PAR 6100Bomb Calorimeter. Properties of the amendments used are given in Table 2 and can be seen in Fig. 1.

*L. terrestris* (anecic) earthworms were obtained commercially from wormsdirectuk.co.uk to ensure an abundant supply of specimens of similar size and age. They were in good condition (i.e. well hydrated), responsive (determined my assessing their response to a physical stimuli to the anterior), were all clitellate, and had mean masses of 1.7 g (SD: 0.39, n = 372). Earthworms were equilibrated to our laboratory conditions, following Fründ et al. (2010), in a culture made from the same silty clay loam soil (Fosters field, Rothamsted) used in the experiments and fed with Irish Moss Peat, following Spurgeon et al. (2000) at approximately 1 g earthworm^−1^ week^−1^ for more than one week prior to addition to experimental microcosms.

#### 2.2.2 Microcosm experimental design

Experimental microcosms were constructed using polyethene bags and 1 pint (0.57 Litre) plastic drinking cups (Fig. 1). Soil was wetted up to 70% of the water holding capacity and a treatment applied, as described below, before 500 g (dry wt.) of soil was added to each polythene bag. A pin was used to perforate the top of each plastic bag to allow the circulation of air. The bag was placed in the plastic drinking cup to ensure at least 10 cm depth of soil for the earthworms to burrow (Lowe and Butt, 2005). The mass of a single earthworm was determined before it was added to each microcosm at the start of the experiment. This stocking density is below the 3–5 adult worms l^−1^ rate recommended by Lowe and Butt (2005) so it is unlikely that the earthworms were stressed due to a lack of space. Experimental microcosms were arranged in a complete randomised block design in a controlled environment chamber, in constant darkness at 15 °C. Earthworms were removed from the microcosms by destructive sampling and thorough mixing of the soil every 2 weeks for the duration of the experiment to ensure that the removal of each earthworm had an equal impact on the soil structure and the position of the food in each microcosm. Earthworms were washed by submerging them in deionised water, blotted dry, their mass determined, and then returned to the same microcosm.

#### 2.2.3 Microcosm experiment 1: comparing amendments and straw-amendment mixtures

Before earthworms were added to the experimental microcosms, soil was thoroughly mixed with five rates of <1 mm milled farmyard manure, compost, or anaerobic digestate (Table 3), each relating to 0, 2, 4, 6 and 8 g C kg^−1^ soil (13 treatments). Each of these 13 treatments was further amended and thoroughly mixed with <1 mm milled straw at five rates, also relating to 0, 2, 4, 6 and 8 g C kg^−1^ soil. Each of the resulting 65 treatments was replicated four times comprising a total of 260 experimental microcosms (Table 1). No further applications of organic amendments were made to the pots after this initial addition. Every two weeks of the 12 week duration of the experiment the earthworms were removed from the microcosms, their mass determined, and returned. The soil was homogenised each time the earthworm was removed.

#### 2.2.4 Microcosm experiment 2: comparing wheat and barley straw

After earthworms were added to the experimental microcosms and had burrowed into the soil, the microcosms were amended with six rates of either wheat or barley straw milled to <1 mm by adding the straw to the surface of the pot. Every two weeks, when the earthworm was removed and its mass determined, any straw remaining on the surface was mixed in with the soil and then, after the earthworm was returned to the microcosm and burrowed into the soil, a new application was made to the soil surface. Each straw was applied at a rate of 0, 2, 4, 6, 8 and 10 g kg^−1^ month^−1^, resulting in 11 treatments, and replicated four times, resulting in a total of 44 experimental microcosms (Table 1). The experiment was continued for 10 weeks.

#### 2.2.5 Microcosm experiment 3: comparing wheat straw particle size

After the earthworm was added to the experimental microcosms and had burrowed into the soil, the soil was amended with four rates of wheat straw that had either been (i) milled to <1 mm, (ii) milled to <3 mm, (iii) chopped to 1 cm pieces using scissors, or (iv) been chopped with a bale chopper to approximately 10 cm pieces, analogous to the chopping of straw behind a combine harvester. Straw was applied every two weeks for 16 weeks, in the same manner as in Experiment 2 at rates of 0, 2, 4, 6 and 8 g kg^−1^ month^−1^, each replicated four times, resulting in 17 treatments and 68 experimental microcosms (Table 1).

### 2.3 Statistical analysis

All statistical analysis was carried out in Genstat, version 16.2.0.11713. Analysis of Variance (ANOVA) and Fisher's least significant difference test were employed to test significant differences between treatments at a single time point. Repeated Measures ANOVA was used to discriminate between treatments of microcosm experiments when data from all time points was included in the analysis. In all cases normality was checked by inspecting the residual plots and homoscedasticity confirmed using Bartlett's test (P > 0.05).

## 3 Results

### 3.1 Field surveys

**Fig. 2:**
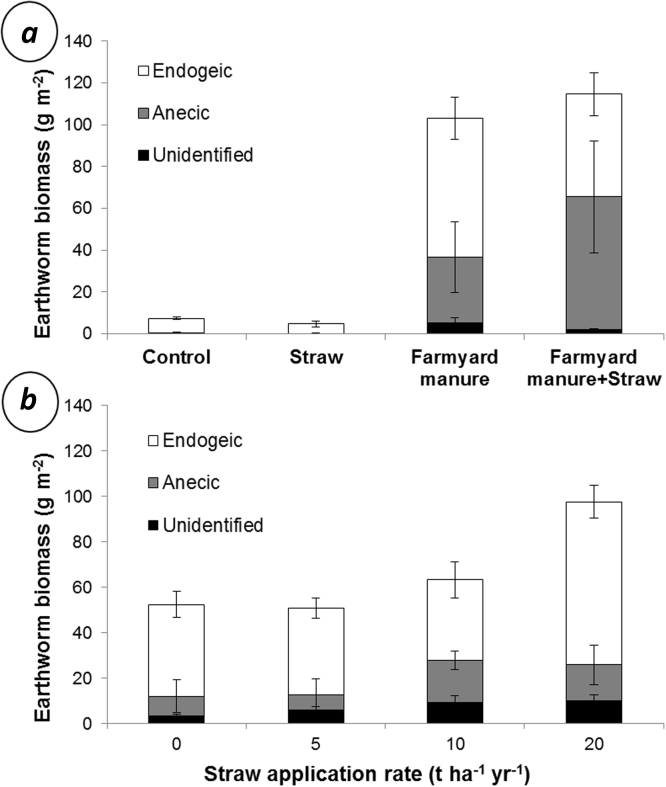
Biomass of endogeic, anecic, and unidentified earthworms determined by surveys of plots on (a) the Broadbalk field experiment and (b) the Long Term Straw Incorporation Experiment at Rothamsted Experimental Farm. Each bar is the average of four replicate plots or subplots with two pseudoreplicate surveys conducted per plot/subplot. Error bars are standard errors of the mean.

Farmyard manure significantly (p < 0.001) increased the biomass of earthworms in the Broadbalk plots (Fig. 2a). This increase was due to a significantly greater biomass and number of endogeic (p < 0.001), anecic (p < 0.05), mature (p < 0.01) and juvenile (p < 0.01) earthworms in the farmyard manure treatments (see Table A1, Table A2). Straw had no significant effect on the earthworm population in the Broadbalk experiment and there were no significant interactions between straw and farmyard manure on earthworm abundance or biomass.

Only the highest rate of straw application resulted in significantly (p < 0.05) greater earthworm abundance and biomass (Fig. 2b) of the Long Term Straw Incorporation Experiment and this was reflected by a significantly (p < 0.05) greater abundance of both juvenile and mature earthworms (see Table A3, Table A4). This difference is largely due to a significantly greater number and biomass (p < 0.01) of endogiec earthworms in the 20 t ha^−1^ treatment. Although we found a significantly greater number of anecic earthworms in the 10 t ha^−1^ and 20 t ha^−1^ treatments, compared to the 5 t ha^−1^ and 0 t ha^−1^ plots, there was no significant difference in the biomass of anecic earthworms between any of the treatments.

Because both earthworm surveys were conducted at different times, they cannot be compared with one another statistically since the results of earthworm surveys are highly dependent on the temperature and moisture of the soil (Eggleton et al., 2009)

### 3.2 Microcosm experiments

Across all three microcosm experiments there was a 92% survival rate over the duration of the experiments (which ranged from 10 weeks to 16 weeks depending on the individual experiment). The high survival rate indicates that the experimental conditions were suitable for culturing the earthworms, even when starvation conditions were imposed in the control treatments. Units in which mortality occurred were excluded from the dataset and treated as missing data during statistical analysis.

#### 3.2.1 Microcosm experiment 1: comparing amendments and straw-amendment mixtures

**Fig. A1:**
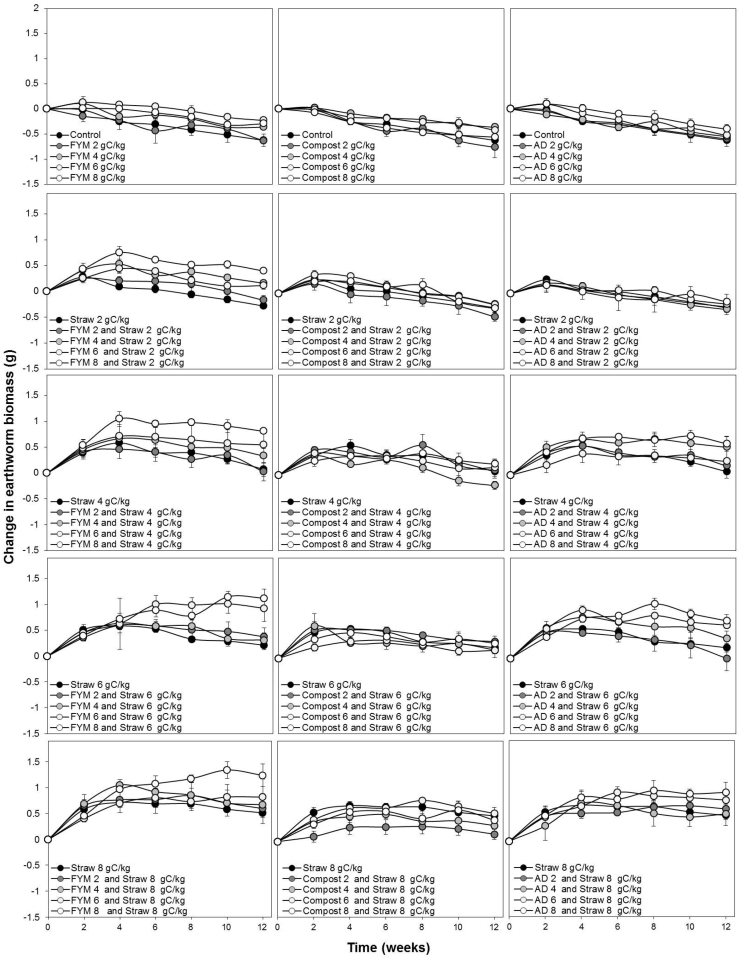
Change in *Lumbricus terrestris* biomass over the course of a 12 week experiment. Earthworms were incubated in bags containing soil amended with straw, manures (farmyard manure; FYM, compost, and anaerobic digestate; AD) and mixtures of straw and manure. Data presented is the mean change in biomass for four replicate earthworms since the start of the experiment. Error bars are standard errors of the mean.

**Fig. 3:**
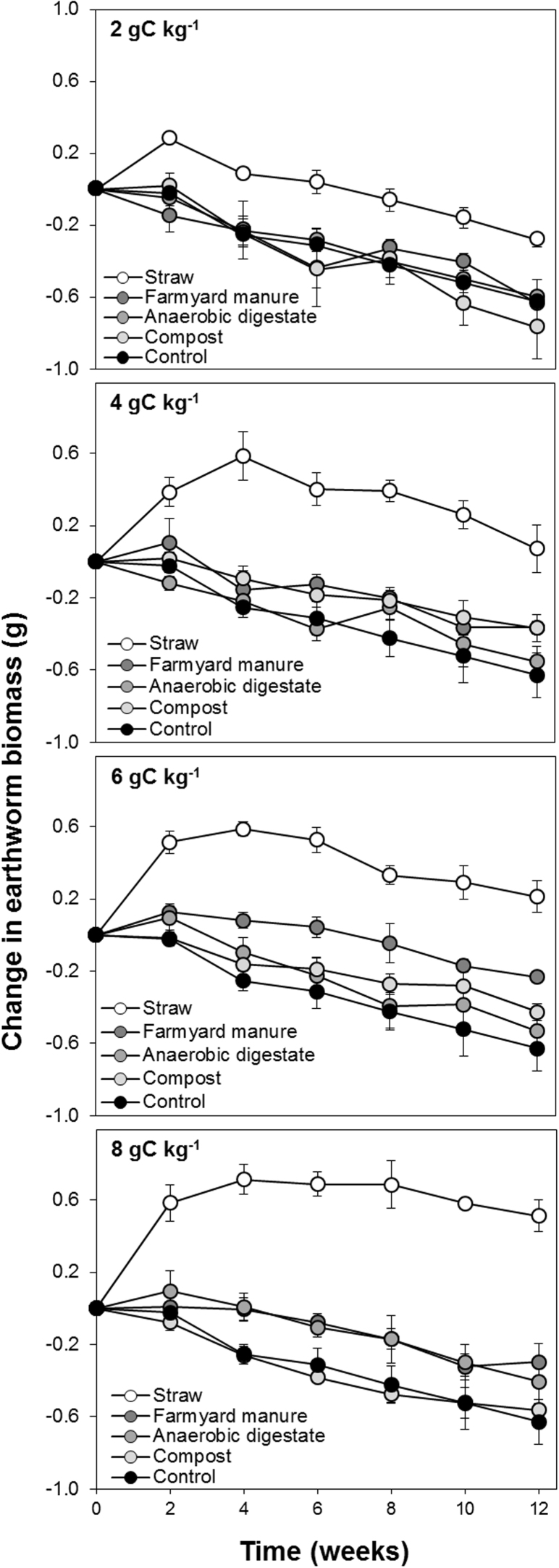
Change in the biomass of *Lumbricus terrestris* earthworms over the course of a 12 week. Either no food (i.e. control treatments), straw, farmyard manure, anaerobic digestate, or compost was added to each microcosm at the start of the experiment at a rate equivalent to 2, 4, 6 and 8 g C kg^−1^. Each data point is the mean of four replicates. Error bars are standard errors of the mean.

The change in earthworm biomass over the 12 week course of the experiment for all 65 treatments treatment is presented in Figure A1 and displayed for selected treatments in Fig. 3. The addition of manures (farmyard manure, compost and anaerobic digestate: p < 0.001), the rate of manure amendment (p < 0.05), and rate of straw amendment (p < 0.001), all significantly affected earthworm biomass during the experiment, with high rates resulting in greater earthworm biomass. The amendments increased earthworm biomass, relative to the unamended control, in the order straw > farmyard manure > anaerobic digestate > compost (Fig. 3).

**Fig. 4:**
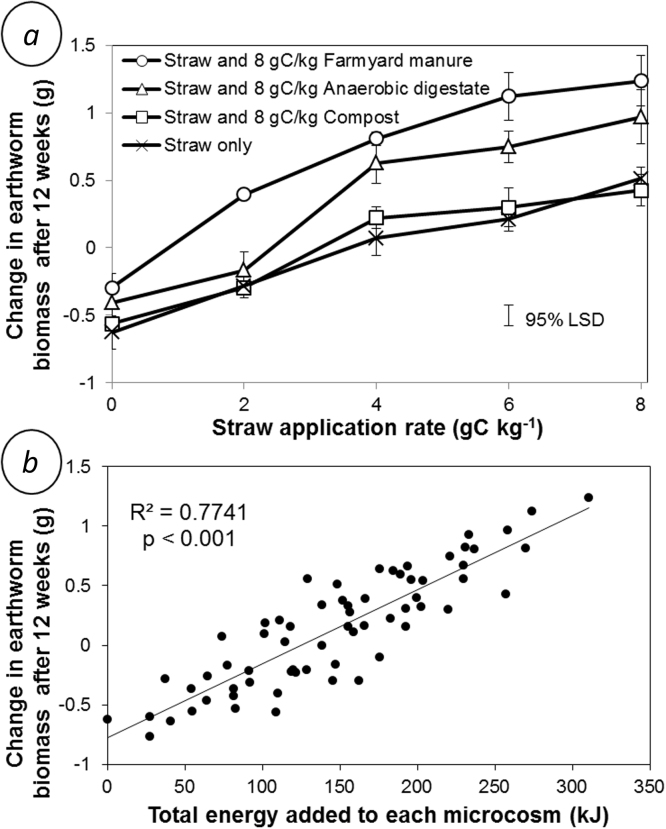
Change in the biomass of *Lumbricus terrestris* earthworms over the course of a 12 week experiment where barley straw and organic amendments (farmyard manure, anaerobic digestate and compost) were added individually and in combination at rates equivalent to 0, 2, 4, 6 and 8 g C kg^−1^. The figure demonstrates (a) the significantly greater change in biomass resulting from farmyard manure and anaerobic digestate applications to earthworms already receiving straw, and (b) the significant positive relationship between the energy of amendments fed to each earthworm and the change in earthworm biomass. Each data point is the mean of four replicates. Error bars are standard errors of the mean.

Straw out-performed all of the other amendments, increasing earthworm biomass by 37% after 12 weeks at the rate of 8 g C kg^−1^, compared to decreases of 17%, 23% and 28% for farmyard manure, anaerobic digestate and compost, respectively (Fig. 3). There was, however, a significant (p < 0.001) interaction between manure rate and straw rate. The positive impact of organic amendments (particularly farmyard manure and anaerobic digestate) on earthworm biomass was greater when applied in combination with straw (Fig. 4a). We found a significant (p < 0.001) positive correlation between the quantity of energy added to the soil within the organic amendments and the resulting change in earthworm biomass over the 12 week duration of the experiment (Fig. 4b) which was stronger (R^2^ = 0.77) than the relationship between%C and change in earthworm biomass (R^2^ = 0.66).

#### 3.2.2 Microcosm experiment 2: comparing wheat and barley straw

The addition of either barley or wheat straw significantly (p < 0.001) increased the biomass of earthworms in the experimental microcosms and earthworm biomass was significantly (p < 0.05) greater when higher rates of straw were applied. However, there was no significant difference in the change in earthworm biomass due to the type of straw applied to the soil. Since the energy contents of these two types of straw are similar (barley straw has 17.0 and wheat straw has 16.4 kJ g^−1^: Table 2) it seems that the energy in each straw is equally accessible to the earthworms.

#### 3.2.3 Microcosm experiment 3: comparing wheat straw particle size

The presence (p < 0.001), rate (p < 0.05), and particle size (p < 0.001) of straw all significantly affected the change in earthworm biomass over the 16 week duration of the experiment (Fig. 6). After 16 weeks, the change in earthworm biomass in the chopped straw or 1 cm straw treatments was significantly (p < 0.05) greater than the control treatments, which saw a decrease in biomass of approximately 0.5 g per earthworm. However, the increase in earthworm biomass due to applying straw cut to 1 cm pieces was not significantly (p > 0.05) different to the increase due to the straw chopped with a bale chopper. Milling the straw to <3 mm particles increased earthworm biomass by 17%, 29%, 36% and 42% when applied at rates of 2, 4, 6 and 8 g kg^−1^ month^−1^, respectively. These increases were significantly (p < 0.05) greater than those observed in treatments where straw was cut to 1 cm (4%, 1%, 7% and 11%) or chopped with the bale chopper (-7%, 6%, 8% and 3%), when applied at rates of 2, 4, 6 and 8 g kg^−1^ month^−1^. Milling to <1 mm particles significantly increased the earthworm biomass by 31%, 50%, 89% and 81% when applied at rates of 2, 4, 6 and 8 g kg^−1^ month^−1^, respectively. These increases in earthworm biomass were significantly (p < 0.05) greater than bale chopping or 1 cm cutting at all rates and significantly (p < 0.05) greater than milling to <3 mm at rates of 6 and 8 g kg^−1^ month^−1^.

## 4 Discussion

### 4.1 *L. terrestris* growth depends on energy content of amendments

We found that straw increased the growth rate of *L. terrestris* to a greater extent than organic manures in the laboratory (Fig. 3). Growth rates could be explained by a positive correlation between the total energy content of a soil amendment and the change in earthworm biomass (Fig. 4b). This correlation is a strong indication that (when all food is ground to the same size and therefore accessible to *L. terrestris*) the calorific value of food is an important factor concerning the growth rate of earthworms. This assertion is supported by observations of laboratory-reared compost earthworms that the nutritional benefits of food is only supplied by cellular mass, that earthworm growth and survival cannot be supported by nutrients alone (Neuhauser et al., 1980), and that paper sludge is a better food source for earthworms than horse manure (Fayolle et al., 1997).

For all the organic manures used in our experiments (farmyard manure, compost and anaerobic disegtate), organisms had already partially used the substrate as an energy source prior to addition to the soil: The manure has passed through the gut of a cow and both the compost and the anaerobic digestate have been metabolised by thermophilic microorganisms under aerobic and anaerobic conditions, respectively. During each of these processes energy is used by the organisms in question (and, in the case of anaerobic digestion by burning the biogas produced). In each case much of the labile energy (i.e. the compounds that are easiest to metabolise) will have been used first. What was left in the final product that was added to the microcosms in this experiment contained less energy and proportionally more recalcitrant energy than the plant material used to generate the manure, compost or digestate. Therefore, even if all the food supplied to the earthworms is accessible (i.e. small enough to ingest), not all of the energy in the food can be metabolised quickly.

The lability of the energy in an amendment depends, not only on the particle size (physical availability), but also on the chemical composition of the substrate (chemical availability). Materials that have a high cellulose/lignin ratio contain more labile energy than materials that have a low cellulose/lignin ratio (McKendry, 2002). Earthworms can produce endogenous cellulase in their gut (Nozaki et al., 2009), which may be responsible for much of the straw degradation, and subsequent increase in *L. terrestris* biomass, observed in our microcosm experiments.

### 4.2 Organic manures support larger earthworm populations in the field than straw, but straw contains more energy

Cereal straw applied to the field plots at a rate commensurate with standard farm practice (∼5 t ha^−1^ yr^−1^) had no significant impact on the size of the earthworm population in the Broadbalk experiment and the Long Term Straw Incorporation experiment, even when applications were made annually for decades (Fig. 2). The observations agree with those of Eriksen-Hamel et al. (2009) who observe no effect of crop residue management on earthworm populations and Stroud et al. (2017) who observe no effect of cover cropping on *L. terrestris* midden abundance. Tian et al. (1993) observed greater earthworm populations when crop residues were surface applied to soils in the humid tropics but populations were negatively correlated with the lignin:nitrogen ratio of the residues, which indicates that the earthworms gain more nutrition from easily digestible residues.

In the Long Term Straw Incorporation experiment only annual applications of wheat straw that were four times the rates harvested (∼20 t ha^−1^ yr^−1^) resulted in an increase (86%) in earthworm biomass whereas the annual application of 35 t ha^−1^ of farmyard manure increased the earthworm biomass by 1290% on the Broadbalk experiment. Assuming 25% dry matter (Powlson et al., 2012) and an energy content of 12.5 kJ g^−1^ (Table 2), 35 t ha^−1^ farmyard manure provides approximately 109 GJ ha^−1^ of energy to the soil, whereas 20 ha^−1^ of wheat straw provides approximately 279 GJ ha^−1^, assuming 85% dry matter (Powlson et al., 2008) and an energy content of 16.4 kJ g^−1^ (Table 2). Our field observations indicate that although the long term incorporation of very high quantities of straw is capable of increasing earthworm populations, application rates commensurate to standard farm practice do not appear to have any impact on the size of the earthworm community and that, per kJ added to the soil, farmyard manure applications are a more efficient way of stimulating earthworm growth.

### 4.3 Organic manure/straw mixtures reveal a synergistic interaction in microcosm experiments, but not under field conditions

We show (Fig. 4a) that the combination of straw with manures (farmyard manure and anaerobic digestate) resulted in the farmyard manure and anaerobic digestate increasing *L. terrestris* biomass more than when manures were applied without straw. This synergistic interaction could occur due to both the straw and manure containing compounds or elements that only provide a benefit to growth when ingested together. Alternatively, the presence of a mixture of amendments may have accelerated the rate of microbial decomposition and thus increased the lability of the energy in the amendments to the earthworm, based on the idea that a greater diversity of organic inputs to soils accelerates residue decomposition (Cong et al., 2015, McDaniel et al., 2014). Despite this significant interaction between crop residues and manures in microcosms, these interactions could not be confirmed in the field. Although we found a greater earthworm biomass in the plot of the Broadbalk field experiment that received both straw and farmyard manure, compared to the manure-only plot (Fig. 2a), this interaction was not statistically significant.

### 4.4 Milling straw appears to result in a more accessible energy source for earthworms

Although there were no significant differences in *L. terrestris* growth in treatments where straw was chopped to 1 cm pieces and treatments in which straw was chopped to ∼10 cm stalks, milling the straw to <3 mm did accelerate growth, and this growth rate was further increased by milling to <1 mm (Fig. 6). The beneficial effect of reducing the particle size of food for earthworm consumption on growth rate has been observed in both organic manures (Lowe and Butt, 2003) and crop residues (Boström and Lofs-Holmin, 1986). Lowe and Butt (2003) showed that the milling of separated cattle solids to < 1 mm increased the mass of *Allolobophora chlorotica* and *L. terrestris* compared to unmilled controls by 185 and 54%, respectively after 18 weeks incubation. Boström and Lofs-Holmin (1986) showed that reducing the size of barley straw and roots from 10 mm to 0.2–1 mm resulted in increases in the growth rate of *Aporrectodea caliginosa*, and that a further reduction to < 0.2 mm resulted in even greater growth rates. Our field observations indicate that earthworms are seemingly unable to ingest straw applied to the soil as long stalks and were thus unable to access the majority of the calories in this food source directly. Therefore, we hypothesise that the incorporation of crop residues with smaller particle size may directly result in a short-term increase in the biomass of *L. terrestris* in the field.

Whalen and Parmelee (1999) recorded *L. terrestris* growth rates to be much lower in the field, compared to the laboratory, despite similar moisture and temperature conditions. Since the food supplied to their laboratory-reared earthworms was first crushed into 2 cm fragments (Whalen and Parmelee, 1999), this may have resulted in particle sizes that *L. terrestris* was able to ingest. Eriksen-Hamel et al. (2009) noted that the incorporation of corn or barley residues in a sandy or clayey soil, respectively, did not significantly affect earthworm biomass in the field. However, when intact soil cores from these field plots were brought into the laboratory, the plots that were subjected to minimum tillage operations (harrowing or chisel ploughing) resulted in the greatest earthworm biomass response to residue application, compared to cores from conventional tillage (moldboard plough/disk harrow) or no tillage plots. The authors suggest that the minimum tillage operations may have reduced the particle size of the residues and made them more palatable to earthworms. Minimum tillage operations also mix straw with soils and provide better substrate distribution in the top few centimetres of the soil compared to ploughing, which buries a mat of straw at depth and is associated with reductions in anecic earthworm biomass (Chan, 2001).

### 4.5 Reducing the particle size of straw applied to soil in the field may increase L. terrestris populations

Approximately 850 Tg of wheat residues alone are produced every year, globally (Talebnia et al., 2010) which represents a considerable energy resource (3872 TWh: more than the entire UK annual energy consumption) and our data indicates that applying these residues to the soil has little impact on the populations of earthworms, an important soil ecosystem engineer. The long-term addition of straw to the soil is however, linked to increased levels of labile C which in turn is correlated with increase aggregate stability and water infiltration (Blair et al., 2006). While we have demonstrated that milling crop residues and applying them to soils in the laboratory does seem to considerably increase the growth rates of *L. terrestris* reared in microcosms, there are several barriers to applying this knowledge in the field to increase earthworm populations in arable soils.

**Fig. 5:**
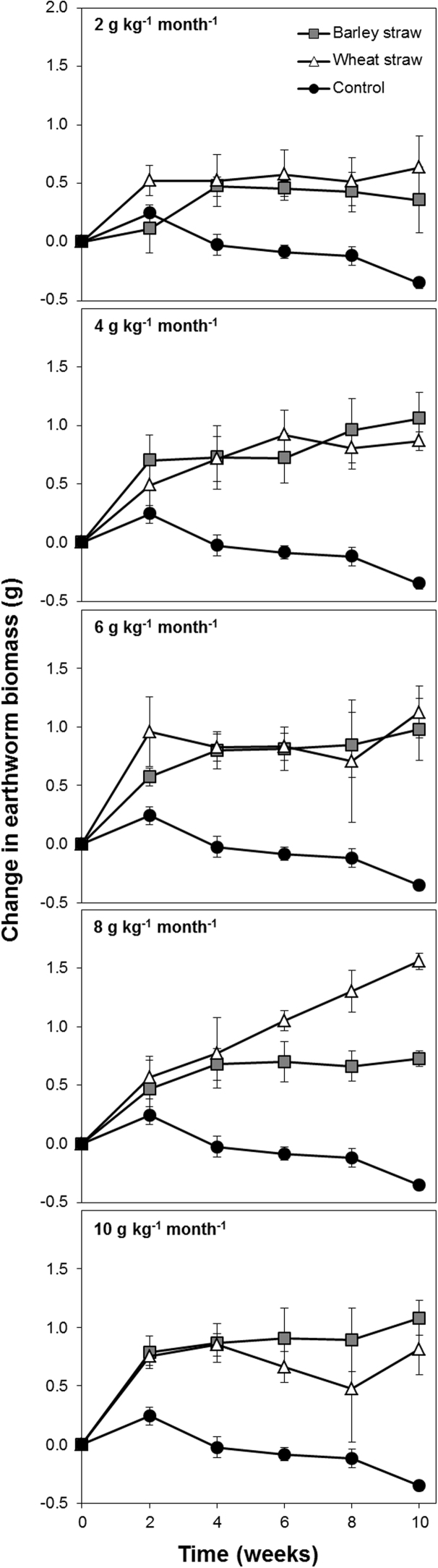
Change in the biomass of *Lumbricus terrestris* earthworms over the course of a 10 week microcosm experiment are receiving no food (i.e. control treatments), wheat straw or barley straw at a rate of 2, 4, 6, 8 or 10 g kg^−1^ week^−1^ applied to the surface of the microcosm. Each data point is the mean of four replicates. Error bars are standard errors of the mean.

**Fig. 6:**
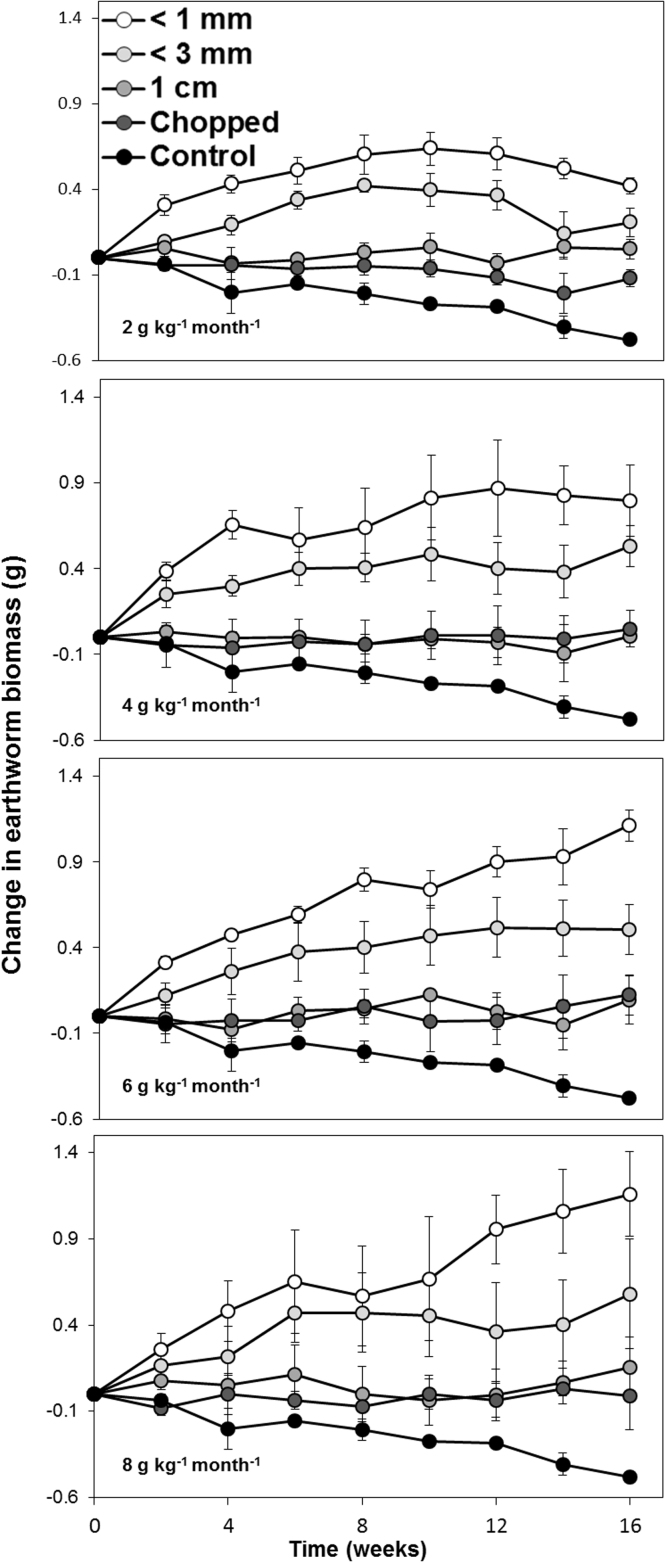
Change in the biomass of *Lumbricus terrestris* earthworms over the course of a 16 week microcosm experiment are receiving no food (i.e. control treatments) or wheat straw with particle size < 1 mm, < 3 mm, 1 cm or chopped to pieces approximately 10 cm in length applied to the surface of microsocms every two weeks at a rate equivalent to 2, 4, 6 or 8 g kg^−1^ month^−1^. Each data point is the mean of four replicates. Error bars are standard errors of the mean.

Milling straw requires a significant input of energy and thus has a financial cost associated with it. Mani et al. (2004) compared the energy required to mill barley and wheat straw using a hammer mill and found that while they were similar, wheat straw required slightly less energy, which is consistent with our anecdotal observations that wheat straw appears to be more brittle. Considering that we observed no significant difference between the barley straw and wheat straw on the growth rate of *L. terrestris* (Fig. 5), and that the total energy content of both straws was similar (Table 2), we propose that either residue is a suitable candidate for field applications. Based on an application rate of 5 t ha^−1^ and an energy requirement of 37 kWh t^−1^ to mill wheat straw at 8.3% moisture content through a 1.6 mm screen (Mani et al., 2004), the energy investment to mill all the wheat straw harvested from a field would be approximately 185 kWh ha^−1^, or 666 MJ ha^−1^. This value compares with an estimated 100–1000 MJ ha^−1^ used to plough arable soils (Bailey et al., 2003, Patterson et al., 1980). If the surface application of straw reduced to < 1.6 mm by a hammer mill (perhaps attached to a combine harvester) increased earthworm populations to the extent that their activities negated mechanical cultivations due to their beneficial soil biological engineering (Bender et al., 2016) then crops of similar yield could potentially be grown with a lower input of energy and labour.

Although our laboratory experiments have revealed that milling crop residues can result in rapid accelerations in growth rate of individual *L. terrestris* earthworms in microcosms containing a single macroinvertebrate, it will be difficult to sustain this level of growth in the field because the milled residues have a higher surface area and will likely be metabolised by the entire soil biological community much more quickly than chopped straw. It may therefore be appropriate to apply milled straw to the field in staged applications throughout the year; applying greater quantities when earthworms are most active. Returning milled residues with multiple applications would likely increase the energy expended and may increase soil compaction by increasing the number of tractor passes. Our future experiments will focus on determining whether staged applications of milled straw can increase earthworm populations in the field and whether this practice can sustainably be incorporated into arable agricultural practice.

## Figures and Tables

**Table A1:** Biomass of earthworms (g m^−2^) determined by surveys of plots on the Broadbalk field experiment at Rothamsted Experimental Farm where wheat straw, farmyard manure (FYM) or a combination of both have been applied annually. Each value is the average of four replicate subplots with two pseudoreplicate surveys conducted per subplot. Standard errors of the mean are provided after each value.

Treatment	Control		Straw		FYM		FYM and straw	
Juvenile *Aporrectodea longa*	0.0	±0.0	0.0	±0.0	4.9	±4.9	9.4	±5.4
Adult *Aporrectodea longa*	0.0	±0.0	0.0	±0.0	0.0	±0.0	0.0	±0.0
Juvenile *Lumbricus terrestris*	0.0	±0.0	0.0	±0.0	4.0	±4.0	10.7	±3.8
Adult *Lumbricus terrestris*	0.0	±0.0	0.0	±0.0	8.7	±8.7	35.6	±29.9
Anecic Juvenile	0.0	±0.0	0.0	±0.0	5.2	±3.6	2.3	±2.3
Anecic bodypart	0.0	±0.0	0.0	±0.0	8.6	±5.0	5.4	±5.4
Juvenile *Aporrectodea caliginosa*	0.0	±0.0	0.8	±0.6	3.3	±1.0	5.2	±1.9
Adult *Aporrectodea caliginosa*	0.0	±0.0	0.0	±0.0	11.7	±5.1	10.7	±5.1
Juvenile *Allolobophora chlorotica*	3.8	±1.8	2.7	±0.4	17.5	±4.4	10.0	±2.6
Adult *Allolobophora chlorotica*	3.2	±2.1	1.1	±1.1	33.7	±5.9	23.1	±3.0
Juvenile *Aporrectodea rosea*	0.0	±0.0	0.0	±0.0	0.0	±0.0	0.0	±0.0
Adult *Aporrectodea rosea*	0.0	±0.0	0.0	±0.0	0.0	±0.0	0.0	±0.0
Juvenile *Lumbricus rubellus*	0.0	±0.0	0.0	±0.0	0.0	±0.0	0.0	±0.0
Adult *Lumbricus rubellus*	0.0	±0.0	0.0	±0.0	0.0	±0.0	0.0	±0.0
Unidentified juvenile	0.4	±0.3	0.0	±0.0	1.7	±0.7	1.2	±0.6
Unidentified bodypart	0.0	±0.0	0.1	±0.1	3.7	±2.1	1.0	±0.6
Total biomass	7.4	±0.9	4.7	±1.4	103.2	±24.6	114.6	±17.8
Total anecic biomass	0.0	±0.0	0.0	±0.0	31.5	±16.9	63.4	±26.7
Total endogeic biomass	7.0	±0.7	4.6	±1.5	66.3	±9.9	49.0	±10.2
Total epigeic biomass	0.0	±0.0	0.0	±0.0	0.0	±0.0	0.0	±0.0
Total unidentified biomass	0.4	±0.3	0.1	±0.1	5.4	±2.3	2.2	±0.4
Total mature biomass	3.2	±2.1	1.2	±1.0	66.4	±20.7	75.9	±24.4
Total juvenile biomass	4.2	±1.6	3.5	±0.5	36.8	±10.2	38.7	±9.1
% juveniles	64.2	±21.2	73.0	±15.7	40.1	±10.1	43.6	±8.0

**Table A2:** Earthworm abundance (number m^−2^) determined by surveys of plots on the Broadbalk field experiment at Rothamsted Experimental Farm where wheat straw, farmyard manure (FYM) or a combination of both have been applied annually. Each value is the average of four replicate subplots with two pseudoreplicate surveys conducted per subplot. Standard errors of the mean are provided after each value.

Treatment	Control		Straw		FYM		FYM and straw	
Juvenile *Aporrectodea longa*	0.0	±0.0	0.0	±0.0	3.1	±3.1	6.3	±3.6
Adult *Aporrectodea longa*	0.0	±0.0	0.0	±0.0	0.0	±0.0	0.0	±0.0
Juvenile *Lumbricus terrestris*	0.0	±0.0	0.0	±0.0	3.1	±3.1	12.5	±5.1
Adult *Lumbricus terrestris*	0.0	±0.0	0.0	±0.0	3.1	±3.1	9.4	±6.0
Anecic Juvenile	0.0	±0.0	0.0	±0.0	6.3	±3.6	6.3	±6.3
Anecic bodypart	0.0	±0.0	0.0	±0.0	6.3	±3.6	0.0	±0.0
Juvenile *Aporrectodea caliginosa*	0.0	±0.0	6.3	±3.6	18.8	±3.6	31.3	±12.0
Adult *Aporrectodea caliginosa*	0.0	±0.0	0.0	±0.0	46.9	±20.0	28.1	±7.9
Juvenile *Allolobophora chlorotica*	50.0	±28.4	46.9	±7.9	212.5	±72.7	103.1	±38.3
Adult *Allolobophora chlorotica*	18.8	±12.0	6.3	±6.3	131.3	±24.2	109.4	±16.4
Juvenile *Aporrectodea rosea*	0.0	±0.0	0.0	±0.0	0.0	±0.0	0.0	±0.0
Adult *Aporrectodea rosea*	0.0	±0.0	0.0	±0.0	0.0	±0.0	0.0	±0.0
Juvenile *Lumbricus rubellus*	0.0	±0.0	0.0	±0.0	0.0	±0.0	0.0	±0.0
Adult *Lumbricus rubellus*	0.0	±0.0	0.0	±0.0	0.0	±0.0	0.0	±0.0
Unidentified juvenile	12.5	±8.8	0.0	±0.0	40.6	±13.9	21.9	±7.9
Unidentified bodypart	0.0	±0.0	0.0	±0.0	0.0	±0.0	0.0	±0.0
Total biomass	81.3	±16.5	59.4	±12.9	471.9	±80.1	328.1	±50.1
Total anecic biomass	0.0	±0.0	0.0	±0.0	21.9	±9.4	34.4	±11.8
Total endogeic biomass	68.8	±19.4	59.4	±12.9	409.4	±81.9	271.9	±54.1
Total epigeic biomass	0.0	±0.0	0.0	±0.0	0.0	±0.0	0.0	±0.0
Total unidentified biomass	12.5	±8.8	0.0	±0.0	40.6	±13.9	21.9	±7.9
Total mature biomass	18.8	±12.0	6.3	±6.3	187.5	±43.6	146.9	±17.2
Total juvenile biomass	62.5	±22.2	53.1	±9.4	284.4	±68.5	181.3	±44.9
% juveniles	74.0	±15.1	85.0	±8.9	58.9	±9.4	52.0	±7.5

**Table A3:** Biomass of earthworms (g m^−2^) determined by surveys of plots on the Long Term Straw Incorporation field experiment at Rothamsted Experimental Farm where wheat straw has been applied annually at rates equivalent to none, once, twice, and four times the yield of straw the previous year (approximately 0, 5, 10 and 20 t ha^−1^). Each value is the average of four replicate plots with two pseudoreplicate surveys conducted per plot. Standard errors of the mean are provided after each value.

Treatment	Control		Straw		FYM		FYM and straw	
Juvenile *Aporrectodea longa*	0.0	±0.0	0.0	±0.0	0.0	±0.0	0.0	±0.0
Adult *Aporrectodea longa*	0.0	±0.0	0.0	±0.0	0.0	±0.0	0.0	±0.0
Juvenile *Lumbricus terrestris*	3.9	±2.2	4.7	±2.7	6.0	±4.6	15.8	±6.1
Adult *Lumbricus terrestris*	8.8	±7.3	2.7	±2.7	0.0	±0.0	6.8	±6.8
Anecic Juvenile	0.0	±0.0	0.0	±0.0	0.0	±0.0	0.0	±0.0
Anecic bodypart	0.0	±0.0	0.0	±0.0	0.0	±0.0	1.2	±1.2
Juvenile *Aporrectodea caliginosa*	3.2	±1.0	6.0	±4.4	9.2	±3.0	1.9	±1.9
Adult *Aporrectodea caliginosa*	8.4	±5.3	7.6	±1.6	11.1	±5.2	10.6	±3.6
Juvenile *Allolobophora chlorotica*	11.2	±1.5	10.9	±2.6	18.7	±2.6	14.0	±4.3
Adult *Allolobophora chlorotica*	13.8	±9.1	19.8	±3.2	16.6	±3.6	18.3	±0.9
Juvenile *Aporrectodea rosea*	0.5	±0.5	0.7	±0.4	0.6	±0.4	0.0	±0.0
Adult *Aporrectodea rosea*	0.0	±0.0	0.0	±0.0	2.4	±0.1	0.0	±0.0
Juvenile *Lumbricus rubellus*	0.0	±0.0	0.0	±0.0	0.0	±0.0	0.0	±0.0
Adult *Lumbricus rubellus*	0.0	±0.0	0.0	±0.0	0.0	±0.0	0.0	±0.0
Unidentified juvenile	4.2	±1.7	6.0	±2.0	2.7	±2.0	7.7	±1.2
Unidentified bodypart	0.5	±0.5	3.1	±1.3	1.2	±0.5	3.2	±1.6
Total biomass	54.5	±15.9	61.7	±6.4	68.5	±11.2	79.5	±15.5
Total anecic biomass	12.7	±6.5	7.4	±4.8	6.0	±4.6	23.9	±8.5
Total endogeic biomass	37.1	±13.2	45.1	±6.0	58.7	±10.5	44.8	±7.6
Total epigeic biomass	0.0	±0.0	0.0	±0.0	0.0	±0.0	0.0	±0.0
Total unidentified biomass	4.7	±1.6	9.1	±2.3	3.9	±1.7	10.9	±2.4
Total mature biomass	31.5	±13.6	33.3	±3.3	31.3	±8.7	40.1	±8.9
Total juvenile biomass	23.0	±2.5	28.3	±9.2	37.2	±5.4	39.4	±9.0
% juveniles	54.8	±9.1	45.0	±4.9	55.7	±6.3	47.0	±8.5

**Table A4:** Earthworm abundance (number m^−2^) determined by surveys of plots on the Long Term Straw Incorporation field experiment at Rothamsted Experimental Farm where wheat straw has been applied annually at rates equivalent to none, once, twice, and four times the yield of straw the previous year (approximately 0, 5, 10 and 20 t ha^−1^). Each value is the average of four replicate plots with two pseudoreplicate surveys conducted per plot. Standard errors of the mean are provided after each value.

Treatment	Control		Straw		FYM		FYM and straw	
Juvenile *Aporrectodea longa*	0.0	±0.0	0.0	±0.0	0.0	±0.0	0.0	±0.0
Adult *Aporrectodea longa*	0.0	±0.0	0.0	±0.0	0.0	±0.0	0.0	±0.0
Juvenile *Lumbricus terrestris*	6.3	±3.6	9.4	±6.0	6.3	±3.6	9.4	±3.1
Adult *Lumbricus terrestris*	6.3	±3.6	3.1	±3.1	0.0	±0.0	9.4	±3.1
Anecic Juvenile	0.0	±0.0	0.0	±0.0	0.0	±0.0	0.0	±0.0
Anecic bodypart	0.0	±0.0	0.0	±0.0	0.0	±0.0	0.0	±0.0
Juvenile *Aporrectodea caliginosa*	15.6	±6.0	18.8	±10.8	34.4	±14.8	12.5	±12.5
Adult *Aporrectodea caliginosa*	25.0	±11.4	21.9	±6.0	31.3	±10.8	25.0	±8.8
Juvenile *Allolobophora chlorotica*	90.6	±13.9	109.4	±21.9	196.9	±34.0	131.3	±34.4
Adult *Allolobophora chlorotica*	59.4	±38.7	100.0	±15.3	75.0	±15.3	90.6	±6.0
Juvenile *Aporrectodea rosea*	3.1	±3.1	6.3	±3.6	6.3	±3.6	0.0	±0.0
Adult *Aporrectodea rosea*	0.0	±0.0	0.0	±0.0	12.5	±0.0	0.0	±0.0
Juvenile *Lumbricus rubellus*	0.0	±0.0	0.0	±0.0	0.0	±0.0	0.0	±0.0
Adult *Lumbricus rubellus*	0.0	±0.0	0.0	±0.0	0.0	±0.0	0.0	±0.0
Unidentified juvenile	75.0	±34.2	81.3	±18.8	46.9	±30.4	100.0	±18.4
Unidentified bodypart	0.0	±0.0	0.0	±0.0	3.1	±3.1	0.0	±0.0
Total biomass	281.3	±79.8	350.0	±18.4	412.5	±29.8	378.1	±57.4
Total anecic biomass	12.5	±5.1	12.5	±7.2	6.3	±3.6	18.8	±6.3
Total endogeic biomass	193.8	±42.5	256.3	±30.8	356.3	±36.3	259.4	±41.3
Total epigeic biomass	0.0	±0.0	0.0	±0.0	0.0	±0.0	0.0	±0.0
Total unidentified biomass	75.0	±34.2	81.3	±18.8	50.0	±29.8	100.0	±18.4
Total mature biomass	90.6	±49.6	125.0	±15.3	121.9	±10.7	125.0	±5.1
Total juvenile biomass	190.6	±33.2	225.0	±25.5	290.6	±23.6	253.1	±60.7
% juveniles	73.1	±6.0	63.3	±0.8	69.3	±2.3	64.3	±6.2

**Table 1:** An outline of the individual experiments conducted in this investigation.

Experiment	Field/Laboratory	No. of treatments	Factors	No. of replicates	No. of units
Long Term Straw Incorporation Experiment	Field	4	Straw rate 0, 5, 10 and 20 t ha^−1^	4	16
Broadbalk	Field	4	Organic matter type Farmyard manure, straw, mixture, nil	4 a	16
Microcosm experiment 1	Laboratory	65	Organic matter type Straw, farmyard manure, anaerobic digestate, compost Organic matter rate 0, 2, 4, 6 and 8 g C kg^−1^ soil Straw-manure mixtures	4	260
Microcosm experiment 2	Laboratory	11	Straw type Wheat straw, barley straw Straw rate 0, 2, 4, 6, 8 and 10 g kg^−1^ month^−1^	4	44
Microcosm experiment 3	Laboratory	17	Straw particle size < 1 mm, <3 mm, 1 cm and chopped Straw rate 0, 2, 4, 6 and 8 g kg^−1^ month^−1^	4	68

a Subplots are considered here as true replicates.

**Table 2:** Properties of soil amendments used in microcosm experiments.

Soil amendment	%N	%C	C:N	Gross energy (kJ g^−1^)
Barley Straw	0.50 (0.003)	46 (0.09)	92	17.0
Farmyard Manure	2.7 (0.008)	31 (0.04)	11	12.5
Anaerobic Digestate	2.4 (0.013)	42 (0.23)	17	11.5
Compost	1.4 (0.022)	29 (0.88)	21	8.0
Wheat Straw	0.53 (0.003)	45 (0.10)	84	16.4

Mean of three replicate samples. Standard errors in brackets.

**Table 3:** Rates of organic amendment applied in microcosm experiment 1.

Rate gC kg^−1^	Barley Straw g kg^−1^	Farmyard Manure g kg^−1^	Anaerobic Digestate g kg^−1^	Compost g kg^−1^
0	0	0	0	0
2	4.4	6.5	4.8	6.8
4	8.7	13.0	9.6	13.6
6	13.1	19.5	14.4	20.5
8	17.4	26.0	27.3	19.2
